# Expression of TolC and Organic Solvent Tolerance of Escherichia Coli Ciprofloxacin Resistant Mutants

**Published:** 2017

**Authors:** Razieh Pourahmad Jaktaji, Farzaneh Zargampoor

**Affiliations:** a *Faculty of Science and Biotechnology Center, University of Shahrekord, Iran.*; b *Faculty of Science, Shaheed Chamran University, Tehran, Iran.*

**Keywords:** AcrAB-TolC efflux pump, Ciprofloxacin, *E. coli*, Organic solvent tolerance, *TolC*, *marR*

## Abstract

AcrAB-TolC is a major efflux pump in *Escherichia coli*. It was reported that *tolC* is overexpressed and involves in improving the organic solvent tolerance level in *Escherichia coli* *marR* mutants that are resistant to several antibiotics, such as ciprofloxacin. Low and intermediate levels resistance did not improve organic solvent tolerance. Thus, it was decided to measure tolC expression and organic solvent tolerance in high level ciprofloxacin resistant mutants. tolC expression was measured by real time PCR and organic solvent tolerance assay was conducted by counting bacterial colonies on LBGMg agar. Results showed that tolC expression was increased significantly (P<0.05) and organic solvent tolerance was slightly improved in high resistant mutants. It was concluded that high organic solvent tolerance may need higher expression of tolC.

## Introduction

Antibiotic resistance is a major problem worldwide. It is occurred by chromosomal mutation in antibiotic target genes (1). However, high-level resistance needs multiple mutations, especially in genes encoding efflux pump regulator proteins. The major efflux pump in gram negative bacteria like *E. coli* is AcrAB-TolC (2-3). As the name shows this is a three-component pump and each component encodes by separate gene, including *acrA* and *acrB* located in same operon, and *tolC* (4). 

Their expressions are up regulated by homologous proteins, including MarA and SoxS independently. Overexpression of *marA* or *soxS* causes enhanced efflux of antibiotics such as fluoroquinolones and organic solvent tolerance (5-9). Various organic solvents are toxic to most microorganisms due to destructive effect on biological membranes (10). 

Among efflux proteins, TolC, the outer membrane channel claimed to have the most important role in maintaining and improving the organic solvent tolerance level in *E. coli* (11). However, AcrA and AcrB, the periplasmic protein and the inner membrane transporter, respectively are activated in organic solvent tolerant *E. coli* mutants (12). These mutants are reported to have mutations in *marR* (13). This gene codes the repressor of *marA* expression (13). 

In the previous study the organic solvent tolerance as well as *acrA* and *marA* expressions were measured in *E. coli* *marR* mutants (14-15). However, they were not organic solvent tolerant and did not overexpress *acrA*. One reason for that is they were not acquired high resistance to fluoroquinolone antibiotics like ciprofloxacin. Thus, it was decided to measure the organic solvent tolerance and the *tolC* expression in *marR* mutants with high level resistance to ciprofloxacin. 

## Experimental

*Antimicrobial agent, chemicals and microbial strains and media*

Ciprofloxacin was obtained from Sigma, USA. Stock solution was 10 mg/mL. *n*-hexane (Merck, Germany) and cyclohexane (Merck, Germany) were organic solvents used in this study. MG1655 was parent strain. *marR *clones (PM1 and PM2) with high resistance to ciprofloxacin (100 µg/mL) derived from *marR* mutants (C6 and C17) isolated in previous work (Pourahmad Jaktaji and Ebadi, 2013). PM1 and PM2 derived from C6 and C17, respectively harboring an alteration of methionine-74 to threonine in MarR and the 20-base pair tandem duplication in promoter site (marO), respectively. Both clones (PM1and PM2) have a Serine 83 to Leucin mutation in GyrA. C17 showed overexpression of *marA*, but not *acrAB* (14-15). LB broth (Merck, Germany) was used to cultivate strain and mutants. An altered medium called LBGMg agar containing 0.1% glucose, 10 mM MgSO_4 _and 1.5% agar was used for organic 

*Solvent tolerance assay*

*Organic solvent tolerance (OST) assay*

Bacterial strains grown to logarithmic phase were used to prepare serial dilutions in 0.9% NaCl and 5 μL of each dilution spotted on LBGMg agar and allowed to dry as described previously (1). The surface of the medium was overlaid with an organic solvent and incubated at 37 °C for 24 h. Then, the number of colonies per spot was counted on each plate. 

*Expression analysis of tolC by real time PCR method*

The fresh cultures of bacterial strains in LB broth were incubated at 37 °C with shaking at 150 rpm and grown to mid-logarithmic phase for RNA extraction using an RNeasy Mini Kit (Qiagen, Germany) following stabilization in RNA protect bacterial reagent (Qiagen, Germany). Elimination of contaminating genomic DNA was conducted by using RNase-free DNase I according to the manufacturerʹs instruction (Fermentas, Life science research) and the absence of DNA was confirmed by amplification of RNA samples and a positive control (DNA sample). The concentration of total RNA was estimated at OD260 using spectrophotometer (Ultrospec 1100, Amersham Pharmacia Biothech). 

The 2 μg RNA was used as a template in RT-PCR reaction using a RevertAid Reverse Transcriptase kit (Fermentas, Life science research). The cDNAs obtained from reverse transcription were used to quantify the level of *tolC *and *gapA*, as an endogenous reference gene by real time PCR in a Rotor Gene 6000 thermocycler (Corbett Research, Australia) using a SYBR Green kit (Takara, Japan). Primers used for real time PCR are listed in Table 1. Thermal cycling conditions were described in SYBER Green kit instruction. Pfaffl method (ratio of target gene expression, *tolC*, to *gapA *expression) was used to calculate relative gene expression (18). Gene expression data are the mean of triplicate analyses. Statistical analysis of relative expression was done by SPSS version 16. T-test was used for comparison of relative gene expression data.

## Results

Organic solvent tolerance levels vary considerably among species. It was shown that MG1655 grows in the presence of *n*-hexane but not cyclohexane (19). In the previous study a series of mutants were isolated from MG1655 in which organic solvent tolerance levels were nearly the same as MG1655 (19-20). These mutants harbor a mutation in *marOR*. Moreover, clones derived from these mutants with intermediate level resistance to ciprofloxacin also showed just better growth on hexane, but no growth on any hexane-cyclohexane mixture. We decided to improve ciprofloxacine resistance stepwise to gain high resistance clones according to procedure described previously (16). Then, the organic solvent tolerance was measured in these mutants (PM1 and PM2). Results are shown in Table 2. These mutants showed low growth on hexane-cyclohexane medium with ratio of 3-1, respectively. However, no growth was seen in media with higher proportions of cyclohexane. This improvement in organic solvent tolerance may due to overexpression of *marA* in these mutants.

All RNA samples used in this study lack DNA contamination. It was shown by PCR amplification of extracted RNA. 

The suitable annealing temperature for *tolC* amplification was 60. The efficiency of real time PCR reaction and coefficient of determination (r^2^) for *tolC* and *gapA* was approximately 1.96 and 0.98, respectively. The melting curve of two genes showed just one major peak indicating their amplification. Cts (treshhold cycles) values obtained from amplification curves for MG1655 and clones were used for calculation of relative expression. Table 3 shows the *tolC *relative expression in these clones. The T-test analysis showed significant difference between wild type and two mutants (PM1 and PM2) for expression of *tolC *(p < 0.05). This result is in agreement with that obtained for organic solvent tolerance. However, the other two mutants (C6 and C17) did not show significant difference with wild type for expression of *tolC*. PM1 and PM2 are similar for organic solvent tolerance and *tolC* expression.

## Discussion

AcrAB-TolC pump is the main pump in *E. coli* which is responsible for multiple resistance to irrelevant antibiotics and organic solvent tolerance (4). It was explained that the organic solvent tolerance can be improved by overexpression of *acrAB*-*tolC* genes (6, 11). 

**Table 1:** Primers used for real time PCR

**Primer**	**Sequence(5′-3′)**	**Amplicon length per primer pairs (bp)**	**Reference**
tolCF	AAGCCGAAAAACGCAACCT	100	(17)
tolCR	CAGAGTCGGTAAGTGACCATC		
gapAF	ACTTACGAGCAGATCAAAGC	170	(16)
gapAR	AGTTTCACGAAGTTGTCGTT		

**Table 2:** Organic solvent tolerance levels of wild type and mutants

**Strain/mutant**	**Growth in the presence of :** a			
	**H**	**H-CH(3:1)**	**H-CH(1:1)**	**H-CH(1:3)**
MG1655	++	-	-	-
C6	++	-	-	-
C17	++	-	-	-
PM1	++	+	-	-
PM2	++	+	-	-

a ++, Excelent growth covered the entire surface of the spots; +, growth; -, no growth; H, n-hexane; CH, cyclohexane; H-CH, mixed solvents with different ratio(vol/vol).

**Table 3:** Relative expression of *tolC* in wild type and mutants

**Stain/mutant**	**Relative expression** a
MG1655	1
C6	1.1
C17	1.1
PM1	2.1
PM2	2.1

a Expression relative to MG1655, mean values from three independent experiments. Figures are the ratio of gene expression between the target gene (*tolC*) and the reference gene (*gapA*). An effect on gene expression was considered significant when the corresponding ratios were > 2 or < 0.6 with a P value of less than 0.05. In all cases the standard deviation was less than 10% of mean.

It was found in previous study that a missense mutation in *marR* causes high organic solvent tolerance level (7). This study aimed to measure organic solvent tolerance and *tolC* expression in mutants with high level resistance to ciprofloxacin. 

It was found that organic solvent tolerance was improved in PM1 and PM2 clones. They were slightly tolerant to cyclohexane. This is consistent with previous finding that a mutation in *marR* is related to organic solvent tolerance. However, it was also found that some mutations in *marR* or *acrR*, encoding the repressor of *acrAB* operon, either alone or in combination cannot improve organic solvent tolerance (1). The location of the mutation in *marR* is different in PM1 and PM2 in comparison to the cyclohexane tolerant mutant in which a change (Arg→Ser) at codon 73 was observed (7). MarA is a positive regulator of AcrAB-TolC efflux pump (5). The introduction of *marA* on low or high copy number plasmids into wild type strain caused cyclohexane tolerance (7). The PM2 clone derived from C17 mutant showed overexpression of *marA*. Thus, it is expected that both PM1 and PM2 may gain increased expression of *marA* and thereby *acrAB*. However, it is possible that the level of expression may not enough for high organic solvent tolerance or other unknown mechanisms are also involved. 

Moreover, it was found that *tolC* was overexpressed in these mutants. This may imply that TolC as a component of AcrAB-TolC pump may be related to increase in organic solvent tolerance. This is consistent with previous work (11). TolC is a component of AcrEF-TolC efflux pump as well. However, it was explained that AcrEF-TolC plays an important role in maintenance of cell division as deletion of *acrEF* does not affect the intrinsic levels of multidrug resistance, but causes cell filamentation (22). It was also suggested that TolC has cooperation with SbmA, an inner membrane transporter protein in acquisition of antibiotic resistance (23). Additionally, it was found that when the expression of *acrAB-tolC* is increased, the expression of other genes code for other pumps, such as acre, *acrF*, *emrE*, *emrD* and *mdfA* is decreased (16). Thus, it seems that AcrAB-TolC efflux pump play a role in organic solvent tolerance. 

In spite of being regulated by the same regulator, MarA, *tolC* expression was reported to be different from *acrAB* in fluoroquinolone resistant *E. coli* mutants (16-17)*. *

Taken together, since TolC participates in different activities, involves in organic solvent tolerance and its expression is different from *acrAB*, it is possible that higher expression of *tolC* is required for high organic solvent tolerance and this may need the activity of other genetic factors. 
